# The role of G proteins in the control of intraflagellar transport

**DOI:** 10.1186/2046-2530-1-S1-P24

**Published:** 2012-11-16

**Authors:** D Huet, SP Perrot, TB Blisnick, PB Bastin

**Affiliations:** 1 Institut Pasteur, France

## 

Intraflagellar transport (IFT) is a mechanism used for formation of cilia and flagella in eukaryotes and little is known about its regulation. We propose that a RAB-like protein termed RABL4 (IFT27), which is associated with IFT complexes could be involved in IFT regulation. Indeed, since small GTPases are known for being key regulators of many trafficking processes. We used different approaches in order to determine the role of RABL4 during flagellum formation in *Trypanosoma brucei*. First, GFP::RABL4 experiments showed that the protein is localized in the cell body, at the basal body and within the flagellum, like all IFT proteins studied so far in trypanosomes. Bi-directional movement of GFP fusion protein was observed in the flagellum. RABL4 silencing demonstrated an essential role in flagellum assembly and revealed a mixture of phenotypes. Some cells do not assemble a flagellum, whereas others produce short flagella filled with material looking like IFT complexes. The structure of these short flagella is severely affected, with disrupted microtubule doublets, mislocalisation of the central pair and impaired PFR (an extra-axonemal structure) construction. The latter one can be excessively large, with numerous layers that affect the shape of the flagellum membrane. We propose that RABL4 could participate to the loading of flagellar precursors into IFT complexes for transport and we are currently analyzing GDP or GTP locked versions of the protein to challenge this hypothesis.

